# Age and Gender Disparities in Adverse Events Following COVID-19 Vaccination: Real-World Evidence Based on Big Data for Risk Management

**DOI:** 10.3389/fmed.2021.700014

**Published:** 2021-07-19

**Authors:** Xiaomo Xiong, Jing Yuan, Minghui Li, Bin Jiang, Z. Kevin Lu

**Affiliations:** ^1^Department of Clinical Pharmacy and Outcomes Sciences, University of South Carolina, Columbia, SC, United States; ^2^Department of Clinical Pharmacy, School of Pharmacy, Fudan University, Shanghai, China; ^3^Department of Clinical Pharmacy and Translational Science, University of Tennessee Health Science Center, Memphis, TN, United States; ^4^Department of Administrative and Clinical Pharmacy, School of Pharmaceutical Sciences, Health Science Center, Peking University, Beijing, China

**Keywords:** vaccine adverse event reporting system, COVID-19, mRNA vaccines, real-world data, real-world study

## Abstract

**Background:** Two coronavirus disease 2019 (COVID-19) vaccines have received emergency use authorizations in the U.S. However, the safety of these vaccines in the real-world remains unknown.

**Methods:** We reviewed adverse events (AEs) following COVID-19 vaccination among adults in the Vaccine Adverse Event Reporting System (VAERS) from December 14, 2020, through January 22, 2021. We compared the top 10 AEs, serious AEs, along with office and emergency room (ER) visits by age (18–64 years, ≥65 years) and gender (female, male).

**Results:** There were age and gender disparities among adults with AEs following COVID-19 vaccination. Compared to younger adults aged between 18 and 64 years, older adults were more likely to report serious AEs, death, permanent disability, and hospitalization. Males were more likely to report serious AEs, death, and hospitalization compared to females.

**Conclusions:** COVID-19 vaccines are generally safe but possible age and gender disparities in reported AEs may exist.

## Background

The coronavirus disease 2019 (COVID-19) pandemic has caused more than 20 million cases and more than 400,000 deaths in the U.S (1). As two COVID-19 vaccines have received emergency use authorizations from the Food and Drug Administration (FDA), there is a hope of ending the pandemic (2–4). Unlike conventionally developed inactivated vaccines, the new type of mRNA vaccines were never marketed before and were expedited with limited clinical trial data, which has raised concerns over their safety (3–5).

Most recently, the Norwegian Medicines Agency (NOMA) reported 29 deaths in older adults occurred shortly after the administration of mRNA COVID-19 vaccines (6), indicating that there may be age disparities in serious AEs and death following COVID-19 vaccination. Therefore, understanding the safety of such vaccines in the real-world (RW) settings is urgently needed. Furthermore, it remains unknown if mRNA vaccines are associated with possible age and gender disparities, as previously reported in a systematic review of disparities in seasonal influenza vaccines (7).

By using national data from Vaccine Adverse Event Reporting System (VAERS), we report the characteristics of AEs and possible age and gender disparities following COVID-19 vaccination.

## Methods

This study used data from VAERS, which is a national post-marketing spontaneous surveillance program for vaccine safety (8, 9). VAERS is co-administered by the Centers for Disease Control and Prevention (CDC) and the Food and Drug Administration (FDA) (8, 9), and it collects information on AEs of vaccines after administration from patients, healthcare providers, vaccine manufacturers, and others (9, 10). AE symptoms in VAERS are coded using the Medical Dictionary for Regulatory Activities (MedDRA), which is a clinically validated, internationally standardized terminology (9, 10). Since June 30, 2017, VAERS labeled a person to have “serious AEs” if any of the following is reported: death, life-threatening illness, hospitalization, existing hospitalization prolonged, permanent disability, and congenital anomaly or birth defect (9–11). VAERS also collects information on office visits and emergency room (ER) visits (11). This study was reviewed and approved by the University of South Carolina Institutional Review Board. Written informed consent for participation was not required for this study in accordance with the national legislation and the institutional requirements.

We reviewed the characteristics of adults aged 18 years or older who reported AEs following COVID-19 vaccination in VAERS from December 14, 2020, through January 22, 2021. Proportional reporting rate per 1,000 people of top 10 reported AEs, serious AEs, and their subtypes (death, life-threatening illness, hospitalization, and permanent disability) were generated along with office and ER visits. We did not report congenital anomaly or birth defect because it occurs only in pregnant women, and there were only a few cases in the VAERS during the study period. To identify possible age and gender disparities in the AEs following COVID-19 vaccination, two strategies were implemented. First, we investigated age- and gender-specific proportional reporting rates for Top 10 AEs, serious AEs and the subtypes following vaccine use. The proportional reporting rate was calculated as the number of a given AE divided by the number of total AE reports following COVID-19 vaccination multiplying 1,000 to report the incident number of the given AE per 1,000 reports. Second, we used a logistic regression model controlling for onset intervals, doses, vaccine manufacturers, and administration types for adjusted odds ratios (AORs) with 95% confidence intervals.

## Results

More younger adults aged between 18 and 64 years reported AEs following COVID-19 vaccination compared to the older adults aged 65 years or older (Table 1). Meanwhile, more females reported AEs than males. Most of the AEs reported occurred within 1 week following the first dose of administration. More AE reports came from the Pfizer-BioNTech's vaccine. Approximately 10% of the reports were serious, and ~2% involved death. More than 5% of the reports involved hospitalization, more than 10% involved office visits, and more than 20% involved ER visits. The top 10 AEs following COVID-19 vaccination were non-serious, including headache, fatigue, dizziness, chills, pyrexia, nausea, pain, injection site pain, pain in extremity, and dyspnoea.

**Table 1:** Characteristics of adults with VAERS reports following COVID-19 vaccination (*N* = 8,976).

**Characteristics**	***N***	**%**
**Age**		
18–64 years	8,207	91.4
65+ years	769	8.6
**Gender**		
Female	7,033	78.6
Male	1,914	21.4
**Onset interval**		
0 day	5,353	61.0
1–7 days	3,145	35.9
8–14 days	179	2.0
≥15 days	93	1.1
**Dose**		
1st dose	7,300	95.9
2nd doses	314	4.1
**Manufacture**		
Pfizer-BioNTech	6,964	77.6
Moderna	2,009	22.4
**Series reports**	1,155	11.8
Death	266	2.7
Life-threatening illness	265	2.7
Permanent Disability	101	1.0
Hospitalizations	698	7.1
**Office visits**	1,194	12.2
**ER visits**	1,998	20.4
**Top 10 AEs**		
Headache	1,977	22.0
Fatigue	1,464	16.3
Dizziness	1,370	15.3
Chills	1,320	14.7
Pyrexia	1,307	14.6
Nausea	1,305	14.5
Pain	1,239	13.8
Injection site pain	904	10.1
Pain in extremity	783	8.7
Dyspnoea	663	7.4

*ER, Emergency room; AE, Adverse event*.

**Figure 1:**
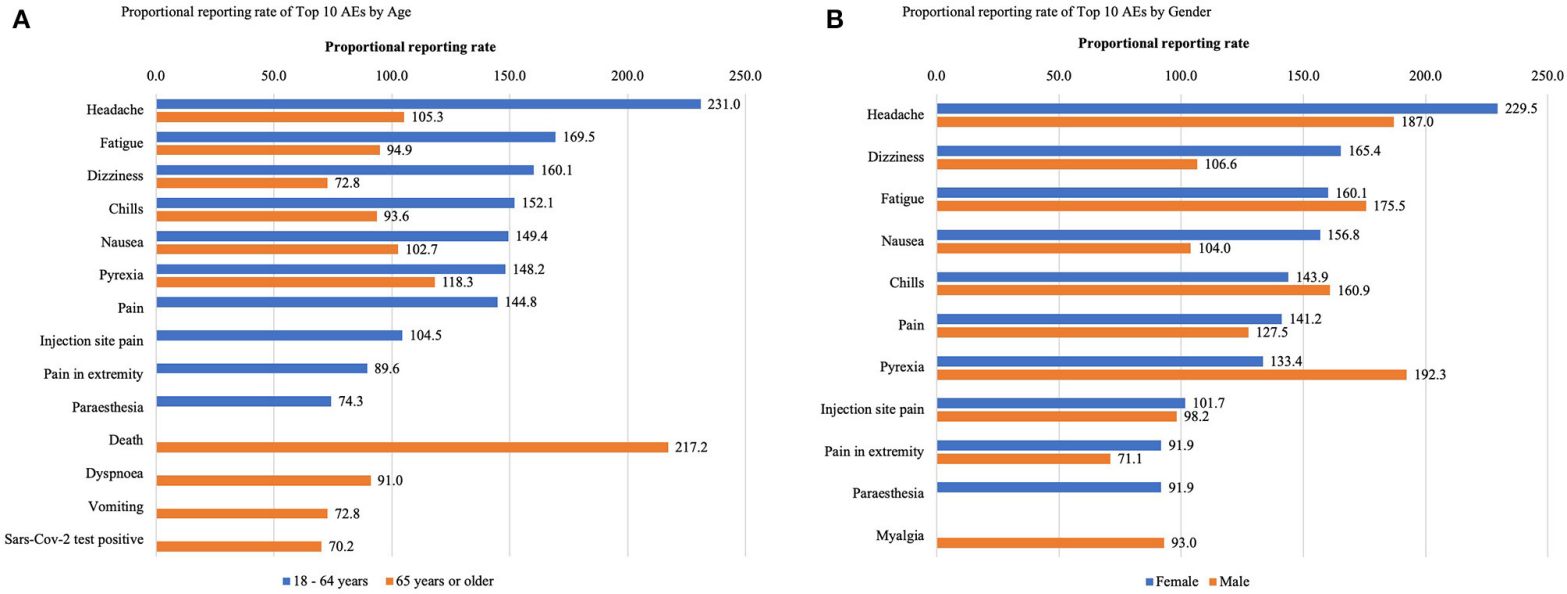
The proportional reporting rate per 1,000 people of the top 10 AEs by **(A)** age and **(B)** gender among adults who had AEs following COVID-19 vaccination. AEs, Adverse events; COVID-19, Corona virus disease 2019; VAERS, Vaccine Adverse Event Reporting System.

**Figure 2:**
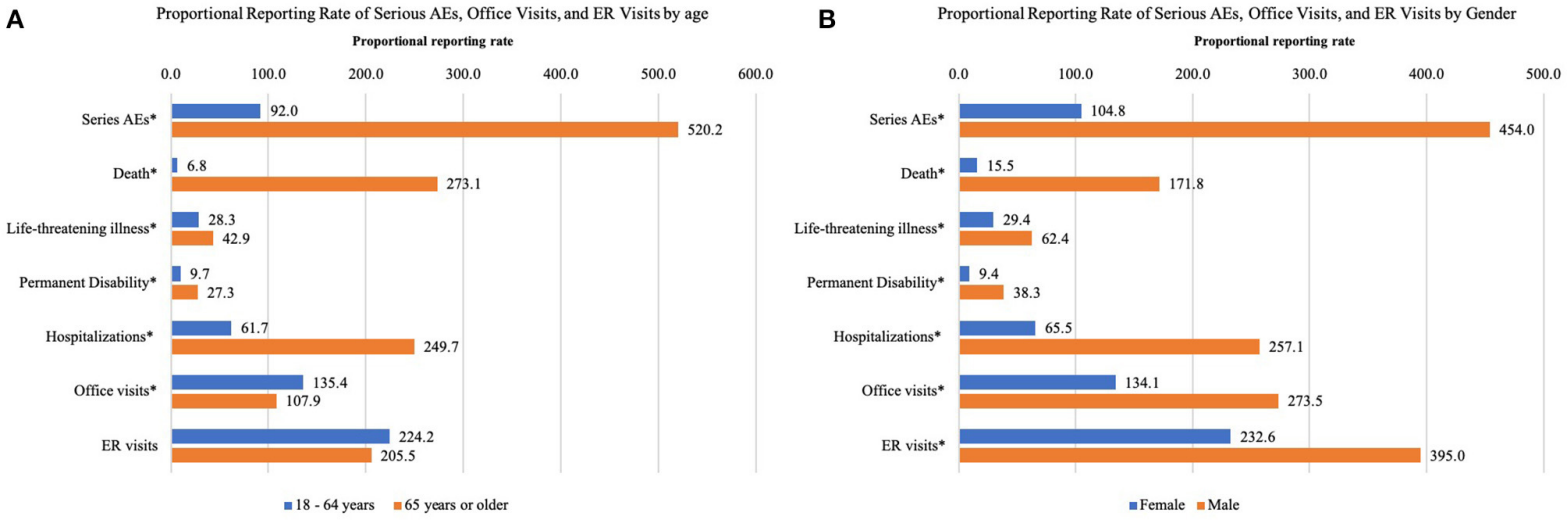
The proportional reporting rate per 1,000 people of serious AEs, office visits, and ER visits by **(A)** age, and **(B)** gender among adults who had AEs following COVID-19 vaccination. Hospitalizations include hospitalization and existing hospitalization prolonged. ER, Emergency room. ^*^Significant at 0.05 confidence level.

In older adults aged 65 years or older, several serious AEs, including death and dyspnoea were among the top 10 AEs but were not in the younger group between 18 years and 64 years (Figure 1A). The proportional reporting rate of serious AEs and the subtypes were significantly higher in older adults than in younger adults, with the exception of ER visits (Figure 2A). As for gender, the top 10 AEs following COVID-19 vaccination were similar between males and females (Figure 1B). However, the proportional reporting rate of serious AE reports and their subtypes, along with office and ER visits, were significantly higher in males than in females (Figure 2B).

**Figure 3:**
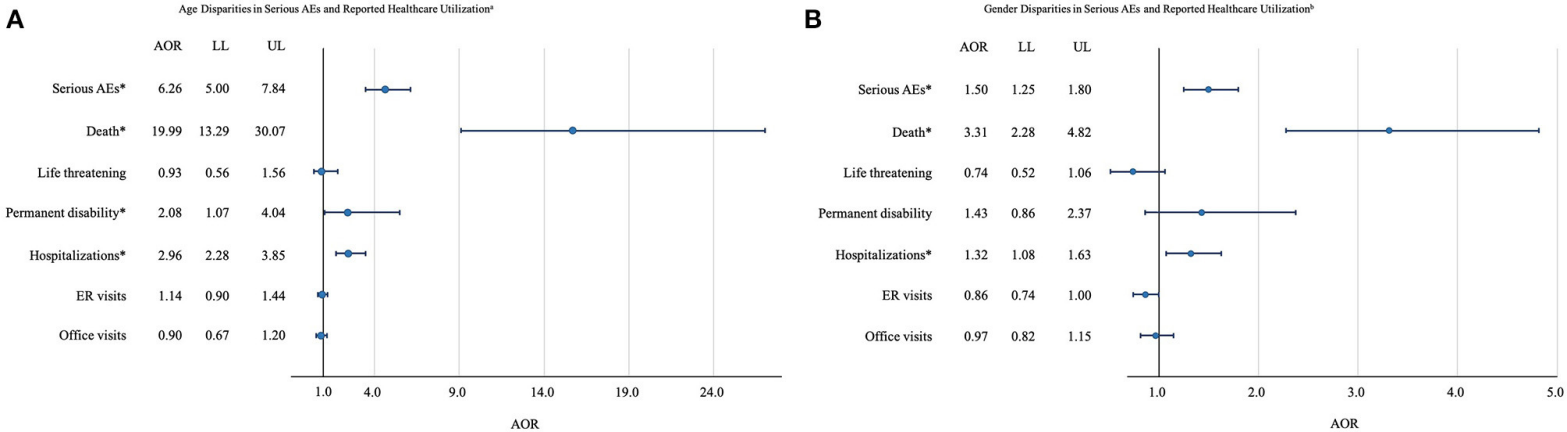
**(A)** Age disparities and **(B)** gender disparities in serious AEs, ER visits, and office visits among adults who had AEs following COVID-19 vaccination in VAERS. The circle represents the point estimate of AOR. The length of the line represents the 95% CI of the AOR. Hospitalizations include hospitalization and existing hospitalization prolonged. AEs, Adverse events; AOR, Adjusted odds ratio; LL, Lower limit; UL, Upper limit; CI, Confidence interval; ER, Emergency room; COVID-19, Coronavirus disease 2019; VAERS, Vaccine Adverse Event Reporting System; ^a^Adults aged 65 years or older vs. adults aged between 18 and 65 years. ^b^Males vs. females. ^*^Significant at 0.05 confidence level.

Results of logistic regression models (Figures 3A,B) showed that compared to younger adults, older adults were more likely to report serious AEs (AOR: 6.26; 95% CI: 5.00–7.84), death (AOR: 19.99; 95% CI: 13.29–30.07), permanent disability (AOR: 2.08; 95% CI: 1.07–4.04), and hospitalization (AOR: 2.96; 95% CI: 2.28–3.85). Besides, compared to females, males were more likely to report serious AEs (AOR: 1.50; 95% CI: 1.25–1.80), death (AOR: 3.31; 95% CI: 2.28–4.82), and hospitalization (AOR: 1.32; 95% CI: 1.08–1.1.63).

## Discussion

This study is the first safety report on age and gender disparities of COVID-19 vaccines based on national safety data. We found that the most frequent AEs following COVID-19 vaccination were non-serious, and most of the AEs occurred within 1 week of the administration. Moreover, more than 20% of adults who reported AEs following COVID-19 vaccination had ER visits, and ~7% of them involved hospitalization. Age disparities were found in the top 10 AEs, as well as serious AEs and their subtypes, which were not reported in the previous RCTs. Our results indicate that several serious AEs (e.g., death) and new development of COVID-19 infection (measured by SARS-COV-2 test positive following vaccination) are among the top 10 AEs in older adults, but not in the younger population aged between 18 and 64 years. These results suggest that older frail adults have a higher proportional reporting rate of serious AEs and a lower rate of office visits, and that males have a higher proportional reporting rate of serious AEs, as well as office and ER visits compared with females.

Logistic regression to compare serious AEs, office visits, and ER visits among adults who had AE following COVID-19 vaccination showed age and gender disparities. The AOR of death of older adults was 19.99 compared to the younger group of 18 and 64 years. Compared with the commonly used approaches of crude reporting odds ratios and crude proportional reporting ratios for surveillance data, using logistic regressions could control for possible confounding factors (12). Results show that older adults were more likely to report serious AEs, death, permanent disability, and hospitalization compared to the younger counterparts. The higher prevalence of death in the older population might be associated with their higher all-cause death rates (13). These older adults are often frail people with serious underlying health conditions and users of medications and polypharmacy (13). Certain vaccine-disease and vaccine-drug interactions might have contributed to or have worsened the outcomes of these older frail adults. However, considering the higher prevalence of serious AEs and death following COVID-19 vaccination, caution should be used when vaccinating older adults to prevent possible fatal events and serious AEs.

Our results show that more females report AEs following COVID-19 vaccination compared to males. In addition, males are more likely to have serious AEs, hospitalizations, and death. However, in 2019, the unadjusted OR of all-cause mortality of males compared with females was 1.12, which shows males have a higher mortality rate in the general population (14). Thus, additional studies are warranted to determine if vaccines pose additional mortality risks for males.

Our results are consistent with age and gender disparities in AEs reported in influenza vaccines. According to a systematic review based on 46 studies, a higher rate of AEs following immunization was reported in females compared with males (7). Also, a study based on two phase three trials reported that compared to younger adults aged between 18 and 64 years, older adults aged 65 years or older had a higher incidence of serious adverse events and deaths following either quadrivalent virus-like particle vaccination or quadrivalent inactivated vaccination (15).

Age and gender disparities in the safety of COVID-19 vaccines found in our study might be related to the different immune responses by different age and gender groups. Males and females have different immune responses to antigens, and there are differences in innate and adaptive immune responses (16). According to Bouman et al., there is a relative suppression of the cellular immune response of the specific immune system in males as compared with females (17). Evidence also shows that compared to the younger population, the older population has a lower ability to establish an effective response to vaccination (18). Specifically, a study by Müller et al. found that there was a lower frequency of neutralizing antibodies in the older population following BNT162b2 vaccination compared to the younger population (19). Different immune responses by different age and gender groups might relate to the strength of immunity so that there were age and gender disparities in AEs following COVID-19 vaccination (20).

However, our study had several limitations. First, as of January 22, 2021, the majority of the vaccination population (82.6%) completed only the first dose by the time of the study (21, 22). The second dose might pose different AEs risks and data are currently limited in the VAERS. Second, it has been a short time since the vaccines were approved for use and the long-term effects remain unknown. Finally, due to the lack of data in the original dataset, there were only a few confounding factors available for adjustment in the regression models. Therefore, we were not able to control for other potential confounding factors, and no causality could be drawn in this study. No prior research on COVID-19 vaccines was able to control for confounding factors, and this study provides evidence for possible age and gender disparities on important safety measures after controlling for potential confounding factors.

## Data Availability Statement

Publicly available datasets were analyzed in this study. The data can be found at: https://vaers.hhs.gov/.

## Ethics Statement

The studies involving human participants were reviewed and approved by the University of South Carolina Institutional Review Board. Written informed consent for participation was not required for this study in accordance with the national legislation and the institutional requirements.

## Author Contributions

XX, BJ, and ZL: concept and design. XX: acquisition, analysis, and interpretation of data. XX, JY, ML, and ZL: drafting of the manuscript. XX, JY, ML, BJ, and ZL: critical revision of the manuscript for important intellectual content. XX: statistical analysis. ZL: administrative, technical, material support, and supervision. All authors contributed to the article and approved the submitted version.

## Conflict of Interest

The authors declare that the research was conducted in the absence of any commercial or financial relationships that could be construed as a potential conflict of interest.
